# Learning to perform a new movement with robotic assistance: comparison of haptic guidance and visual demonstration

**DOI:** 10.1186/1743-0003-3-20

**Published:** 2006-08-31

**Authors:** J Liu, S C Cramer, DJ Reinkensmeyer

**Affiliations:** 1 Department of Mechanical and Aerospace Engineering, University of California, Irvine, CA, USA; 2 Department of Neurology, and Department of Anatomy and Neurobiology, University of California, Irvine, CA, USA; 3 Department of Biomedical Engineering, University of California, Irvine, CA, USA

## Abstract

**Background:**

Mechanical guidance with a robotic device is a candidate technique for teaching people desired movement patterns during motor rehabilitation, surgery, and sports training, but it is unclear how effective this approach is as compared to visual demonstration alone. Further, little is known about motor learning and retention involved with either robot-mediated mechanical guidance or visual demonstration alone.

**Methods:**

Healthy subjects (n = 20) attempted to reproduce a novel three-dimensional path after practicing it with mechanical guidance from a robot. Subjects viewed their arm as the robot guided it, so this "haptic guidance" training condition provided both somatosensory and visual input. Learning was compared to reproducing the movement following only visual observation of the robot moving along the path, with the hand in the lap (the "visual demonstration" training condition). Retention was assessed periodically by instructing the subjects to reproduce the path without robotic demonstration.

**Results:**

Subjects improved in ability to reproduce the path following practice in the haptic guidance or visual demonstration training conditions, as evidenced by a 30–40% decrease in spatial error across 126 movement attempts in each condition. Performance gains were not significantly different between the two techniques, but there was a nearly significant trend for the visual demonstration condition to be better than the haptic guidance condition (p = 0.09). The 95% confidence interval of the mean difference between the techniques was at most 25% of the absolute error in the last cycle. When asked to reproduce the path repeatedly following either training condition, the subjects' performance degraded significantly over the course of a few trials. The tracing errors were not random, but instead were consistent with a systematic evolution toward another path, as if being drawn to an "attractor path".

**Conclusion:**

These results indicate that both forms of robotic demonstration can improve short-term performance of a novel desired path. The availability of both haptic and visual input during the haptic guidance condition did not significantly improve performance compared to visual input alone in the visual demonstration condition. Further, the motor system is inclined to repeat its previous mistakes following just a few movements without robotic demonstration, but these systematic errors can be reduced with periodic training.

## Background

Stroke is the leading cause of disability in the U.S[1]. Robotic devices are increasingly being used as tools for treating movement deficits following stroke, and other neurologic injuries [2-6]. They are also candidates as tools in other neurological conditions characterized by motor deficits, such as multiple sclerosis or spinal cord injury, as well as for training healthy subjects to perform skilful movements, such as those required for surgery, writing, or athletics [7-9]. A key issue in the development of robotic movement training is the selection of appropriate training techniques – i.e. what pattern of forces should the robot apply to the user to facilitate learning? The present study examined whether the addition of mechanical guidance provided by a robotic device during visuomotor learning of a novel movement path was more effective than visual demonstration alone of the path by the robot. We first review previous studies of robotic guidance, both in rehabilitation and skilled motor learning applications, and then describe the rationale for the present study.

### Robotic guidance in motor rehabilitation

A common technique to address the problem of incorrect movement patterns in motor rehabilitation is to demonstrate the correct movement trajectory by manually moving the patient's limb through it [10]. The premise is that the motor system can gain insight into how to replicate the desired trajectory by experiencing it. For example, a common problem addressed by therapists during rehabilitation after stroke is that patients perform arm movements with abnormal kinematics. Patients might elevate the shoulder in order to lift the arm, or lean with the torso instead of extending the elbow when reaching away from the body [11]. Use of incorrect patterns may limit the ability of patients to achieve higher levels of movement ability, and may in some cases lead to repetitive use injuries. Manual guidance of a patient's limbs may also enhance somatosensory input involved in cortical plasticity [12] and reduce spasticity by stretching [13-16].

Although manual guidance is a common technique in neurologic rehabilitation, it is labor intensive and costly. Therefore, efforts are underway to develop robotic devices to automate this technique. Robotic guidance has been shown to improve motor recovery of the arm following acute and chronic stroke [3,17-21]. However, it is still unclear how the application, advantages, requirements, and other aspects of mechanical guidance compare with other post-stroke training techniques. For example, in a pilot study that compared mechanically guided reaching practice to unassisted reaching practice following chronic stroke, improvements in range and speed of reaching seen with mechanically guided practice were not significantly larger than those seen with unassisted practice [21].

### Robotic guidance in skill training

Haptic guidance has also been explored as a technique for improving interaction with complex human-machine interfaces. For example, a "virtual fixture", or robot-produced constraint [22], could be used to limit the motion of a tool to a desired movement range for applications in surgery or other fine position tasks [23-26]. Haptic assistance has also been used as a technique to control dynamic tasks such as driving [27].

As a "virtual teacher", haptic guidance could encourage subjects to try more advanced strategies of movement. For example, in one study [8], subjects were asked to move then stop a free-swinging pendulum as soon as possible, with a shorter stop time considered a better performance. The optimal strategy for a fast stop was to impulsively accelerate then precisely time and size a second impulse to remove the previously injected energy. Such a strategy requires detailed knowledge of the mechanical properties of the system. A robotic device was programmed to move the subject's hand through this strategy, thereby demonstrating it. Although the subjects' learning curves were not significantly better than subjects who did not receive robotic guidance, perhaps because the optimal strategy was too difficult to master, robotic demonstration encouraged subjects to at least try the optimal strategy on their own. Haptic assistance has also been used as a technique to help learn calligraphy, such as Chinese characters [9].

One of the most comprehensive studies of skill learning with haptic guidance to date examined the ability of healthy subjects to learn a complex trajectory with haptic guidance and/or visual demonstration [7]. A robotic device was used to help the subjects to perform a complex three-dimensional trajectory, which consisted of the summation of three sinusoids at different spatial frequencies, and lasted 10 seconds. Subjects trained by moving the hand along with the robot as it moved along the desired trajectory ("haptic training") with or without vision of the hand, or by simply watching the robot move along the desired trajectory ("visual training"). Subjects significantly improved their performance of the trajectory, both with haptic and visual training, over the course of 15 movement attempts. Haptic training helped in learning to replicate the timing of the trajectory. Visual training resulted in better performance of the shape of the trajectory than haptic training without vision. Haptic training with vision produced similar shape learning when compared with visual training alone

This last result – that haptic training with vision was not better than visual training alone for learning the trajectory shape – is somewhat surprising. A priori, one might expect that the availability of two sources of sensory information would be better than just one for learning a shape. Further, subjects physically practiced the desired trajectory during haptic training compared to visual training, since they moved their hand with the robot along the desired path during both the training condition. Moving the hand along the trajectory would seem to be beneficial for learning the required muscle activity. Clarifying any benefits of adding haptic input to visual input for trajectory learning is clearly important for defining the roles of robotic guidance in a wide range of applications, including rehabilitative movement training.

### Rationale for this study

The major goal of this study was therefore to re-examine whether the addition of haptic information via robotic guidance could help in visuomotor learning of a novel trajectory, compared to visual demonstration of the trajectory alone. We used an experimental protocol similar to Feygin et al. (2002) [7], but altered it in several ways to make it more similar to a rehabilitation context. We used a less complex trajectory that lasted a shorter duration, more similar to the multi-joint trajectories used for many activities of daily living, and more similar to the movements that are repeated as part of post-stroke rehabilitation. We also included a larger number of practice repetitions, matching the duration of a typical therapy session. Finally, we required subjects to try to reproduce the desired trajectory several times in a row following the robotic demonstration. Our goal here was to examine the effect of repeated, unguided practice on ongoing learning, since a common clinical observation is time-dependent decay of gains in movement ability, i.e., that patients often fail to retain what has been achieved without regular therapist intervention.

Although the long-term goal is to better understand the role of mechanical guidance in movement rehabilitation, as a first step, unimpaired subjects were studied in the current investigation. The rationale for studying this population is that it permitted unambiguous separation of learning and performance issues. Specifically, interpreting findings in a subject with stroke would be complicated by deficits in strength, as well as cognitive, language, and attentional domains. These concerns were obviated in the current study by enrolment of only healthy subjects capable of performing the task as instructed. Further, the current study may provide insights into treatment of stroke patients, if one assumes that the motor learning processes present in unimpaired persons are at least partially operative during post-stroke motor learning. Portions of this work have been reported in conference paper format [28].

## Methods

### Experimental protocol

**Figure 1:**
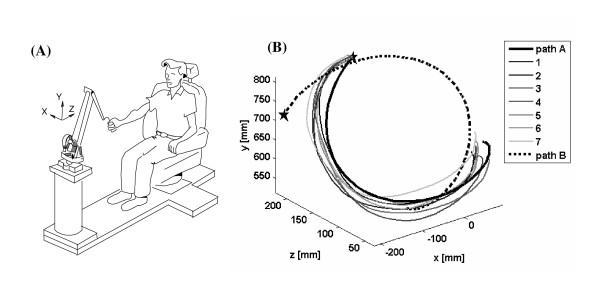
(A) Experimental set up. The subject held the tip of a lightweight robot and tried to move along a desired novel path. (B) 3-D view of the two training paths (path A and B). The star is the start point. The thick line is the desired trajectory. The thin lines are a set of reproduced trajectories in a sample recall phase for one subject for path A.

20 healthy adult subjects (age 18–50) learned to make a novel 3-D path (Figure 1). Subjects held a lightweight haptic robot (PHANToM 3.0, SensAble Technologies, Inc.) with their dominant hand (19 right handed subjects and 1 left handed subject). The protocol was approved by the University of California at Irvine Institutional Review Board, and was in compliance with the Helsinki Declaration. The robot measured hand motion at 200 Hz, and provided haptic guidance along 3-D paths in some conditions (Figure 1). The novel 3-D paths were curves on the surface of a sphere. The following equation was used to transform the movement from spherical coordinates to Cartesian coordinates:

{x=ρ⋅cos⁡θ⋅cos⁡φ+x0y=ρ⋅sin⁡θ⋅cos⁡φ+y0z=ρ⋅sin⁡φ+z0     (1)
 MathType@MTEF@5@5@+=feaafiart1ev1aaatCvAUfKttLearuWrP9MDH5MBPbIqV92AaeXatLxBI9gBaebbnrfifHhDYfgasaacH8akY=wiFfYdH8Gipec8Eeeu0xXdbba9frFj0=OqFfea0dXdd9vqai=hGuQ8kuc9pgc9s8qqaq=dirpe0xb9q8qiLsFr0=vr0=vr0dc8meaabaqaciaacaGaaeqabaqabeGadaaakeaadaGabeqaauaabeqadeaaaeaacqWG4baEcqGH9aqpiiGacqWFbpGCcqGHflY1cyGGJbWycqGGVbWBcqGGZbWCcqWF4oqCcqGHflY1cyGGJbWycqGGVbWBcqGGZbWCcqWFgpGzcqGHRaWkcqWG4baEdaWgaaWcbaGaeGimaadabeaaaOqaaiabdMha5jabg2da9iab=f8aYjabgwSixlGbcohaZjabcMgaPjabc6gaUjab=H7aXjabgwSixlGbcogaJjabc+gaVjabcohaZjab=z8aMjabgUcaRiabdMha5naaBaaaleaacqaIWaamaeqaaaGcbaGaemOEaONaeyypa0Jae8xWdiNaeyyXICTagi4CamNaeiyAaKMaeiOBa4Mae8NXdyMaey4kaSIaemOEaO3aaSbaaSqaaiabicdaWaqabaaaaaGccaGL7baacaWLjaGaaCzcamaabmGabaGaeGymaedacaGLOaGaayzkaaaaaa@7190@

where [x_0 _y_0 _z_0_] is the center of the sphere, ρ is the radius, and Φ and θ are pitch and yaw angles. We set θ and Φ to be linearly related to generate a curve on the sphere:

*Φ *= *c*_1_·*θ *+ *c*_2_

where c_1 _and c_2 _are constants. We varied c_1_, c_2 _and the range of θ to generate two novel paths (Path "A" and "B", Table 1, Figure 1). We chose the path on a sphere because it required learning a novel set of muscle activations, but was not overly complex and was simple to describe mathematically. We considered trying to train a more functional path, such as a reaching path or a feeding motion, but decided against it because such a path would already have been well-learned by the subject. In choosing a novel but simple path, we sought to keep some affinity with what occurs during movement rehabilitation: learning novel muscle activation patterns for relatively simple, multi-joint movements.

**Table 1:** Parameters of the desired paths.

	X_0 _(mm)	Y_0 _(mm)	Z_0 _(mm)	ρ (mm)	c_1_	c_2 _(deg)	θ_start _(deg)	θ_finish _(deg)
Path A	650	200	0	170	-0.25	-60	-225	30
Path B	630	200	0	170	0.2	-15	165	-120

Each subject experienced both a visual training protocol and a haptic training protocol, with the presentation order of the protocols and selection of shapes equally distributed by dividing the subjects into four groups (Table 2). Each training protocol consisted of a sequence of nine cycles. Each of the nine cycles consisted of two phases, a training phase, and a recall phase, with each phase consisting of seven separate movements. During the training phase, the robot demonstrated the desired path to the subject seven times in a row, with the subject just watching the robot (visual training), or moving the arm along with the robot (haptic training). Note that the "haptic training" included both visual and haptic clues, but for simplicity, the term "haptic training" is used in the current report. Immediately following each training phase, there was then a recall phase, during which the subject tried to replicate the path seven times in a row without any assistance from the robot. Therefore, each subject made 63 movements for the visual training (9 × 7) and 126 for the haptic training (63 with the robot guiding the motion and 63 with the robot passive.).

**Table 2:** Path and sequence distribution of haptic training and vision training.

	Number of Subjects	Haptic training protocol	Vision training protocol
Group 1	5	Path A, 1^st ^training set	Path B, 2^nd ^training set
Group 2	5	Path A, 2^nd ^training set	Path B, 1^st ^training set
Group 3	5	Path B, 2^nd ^training set	Path A, 1^st ^training set
Group 4	5	Path B, 1^st ^training set	Path A, 2^nd ^training set

More specifically, in the training phase, the tip of the robot arm was programmed to move along the desired trajectory, using a proportional-integral-derivative position controller. The desired trajectory was equally divided into 1000 positions in check 4 seconds of demonstration time. The proportional, integral, and derivative gains were 0.04 N/mm, 0.00004 N/mm·s, and 0.0012 N·s/mm, respectively. The control command was filtered with a second order Butterworth filter at 40 Hz before sending the command to the robot motors. The parameters θ and ϕ followed half sine wave functions with respect to time, such that their velocities were zero at the beginning and end of movement and maximum midway through the movement. Using this controller, the average tracking error in the training phase between the actual path of the robot tip and desired path was 0.74 (0.04 SD) cm during visual demonstration and 0.85 (0.14 SD) cm during haptic guidance. This indicates that the subjects experienced an accurate version of the desired trajectory during both visual and haptic training.

For the haptic training protocol, subjects were instructed to hold the handle of the robot tip, and move along with the robot, with eyes open. For the visual training protocol, subjects watched the robot tip with their hands resting in their lap. The subjects heard a computerized "beep" when the robot tip was moved to the start point, and another "beep" when the robot tip was moved to the endpoint of the desired path. The robot tip moved back to the start point automatically when the movement was finished, for each of the seven training movements, without the subject holding onto the tip.

During the recall phase that followed each training phase, each subject was asked to reproduce the desired path seven times, with the robot changed to a passive mode. The subject heard a "beep" after the robot tip automatically moved to the start point, a signal for the subject to grasp the handle and begin reproducing the curve. The computer indicated the end of the movement with another "beep" when the total movement time exceeded at least 2 seconds and the velocity was smaller than 3 cm/s. The subject then released the handle and rested with the hand on the lap for approximately 4 seconds as the robot automatically moved back to the start point. After each reproduced movement, the subject was verbally informed of the tracing score, which was inversely proportional to the average tracing error. During the recall phase, the robot was passive and the impedance it presented to the subject was very small: about 0.2 N of backdrive friction and 160 grams of apparent endpoint inertia.

### Data analysis

The robot control loop executed at 1000 Hz, and the position of the robot tip was stored at 200 Hz. To calculate the tracing error, 50 sample points were selected on the desired trajectory by dividing the range of θ associated with the curve into 50 points, and finding the corresponding φ. The tracing error was the minimal distance between each sample point and the reproduced trajectory, averaged across sample points.

A repeated measure ANOVA (using SPSS software) was used to test for an effect of three factors on tracing error: recall cycle number, reach number in each recall cycle, and training condition. Each of these factors was considered a within-subject measure.

## Results

### Path tracing accuracy improved following visual or haptic training

**Figure 2:**
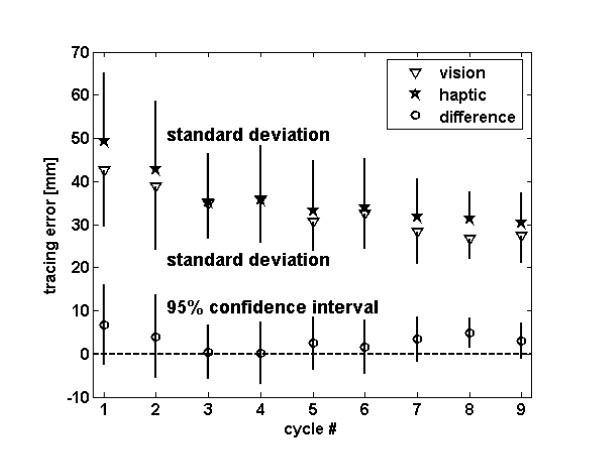
Improvement in tracing error across training cycles. The triangles show the average tracing error after visual demonstration during the recall phase of each cycle. The stars show the average tracing error after haptic guidance during the recall phase of each cycle. The bars show one standard deviation across arms tested. The circles show the difference of tracing error between haptic and visual training, along with the 95% confidence interval for the difference.

The subjects gradually improved their ability to reproduce the novel path. This was true after visual training (i.e. watching the robot move along the path with the hand in the lap) and after haptic training (i.e. moving the hand with the robot along the path with vision). Figure 2 shows the tracing error, averaged across the seven movements in each recall cycle. The tracing error in the first cycle was significantly different from the last cycle (paired t-test, p < 0.001) for both visual and haptic training. Consistent with this, an analysis for presence of a linear contrast following an ANOVA indicated that there was a significant linear dependence (p < 0.001) of tracing error on cycle number.

### Comparison between visual and haptic training

The tracing error after visual demonstration showed a small and non-significant trend towards being smaller than the tracing error after haptic guidance in all 9 cycles (Figure 2, p = 0.09, ANOVA). The 95% confidence intervals of the tracing error difference between the two training techniques included zero in all 9 cycles except the 8^th ^cycle, in which visual demonstration was significantly better than haptic guidance (Figure 2). The 95% confidence interval of the mean difference between the techniques was at most about 25% of the absolute error in the last cycle; thus the techniques were not different by more than 25% in terms of final error, with 95% confidence.

We instructed subjects to move their arms along with the robot during haptic demonstration. To confirm that they did, we analyzed the average force magnitude applied by the robot, and found it was 0.50N (0.08 N SD). During visual demonstration, the average force applied by the robot to move itself alone was similar: 0.43N (0.03N SD). Thus, the subjects indeed moved their arm along with the robot during haptic demonstration, and typically did not "fight" or passively rely on the robot.

### Path tracing error increased when robotic demonstration was withheld

**Figure 3:**
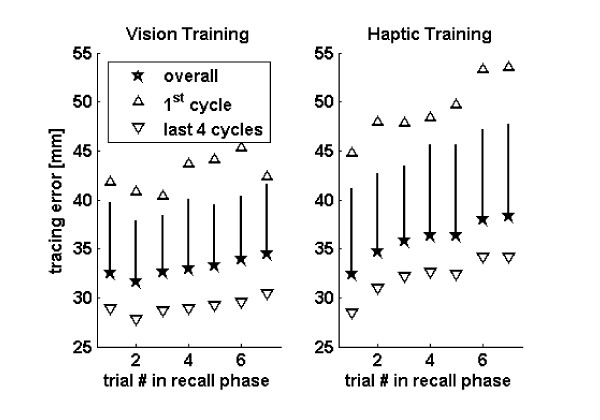
Forgetting during the recall phase of each cycle. The stars show the tracing error after visual (left) or haptic (right) training during the recall phase. The up pointing triangles are the average tracing error in the first cycle across the 20 subjects. The down pointing triangles are the average tracing error in the last 4 cycles across the 20 subjects. The error bars are the standard deviations across the 20 subjects.

Figure 3 shows the tracing error as a function of the reach number during the recall cycle. Whether examining haptic training or visual training, there was an increase in tracing error as the subjects attempted to reproduce the path repeatedly during the recall phase of each cycle (ANOVA, linear contrast, p = 0.002 and p = 0.02 for haptic and visual training, respectively). This process of forgetting was observed in both the early and late stages of the learning (Figure 3). The forgetting process appeared to happen less slowly for visual training, but this effect was not significant (ANOVA, interaction of training technique and trial number within cycle, p = 0.15).

### Tracing error was consistent with a systematic evolution toward an "attractor path"

Visual inspection of the hand paths during the recall phase of each cycle suggested that the increase in trajectory error was due to a systematic and progressive distortion in the hand path, rather than to a random pattern of tracing errors (e.g. Figure 1b). Therefore, we hypothesized that the motor system is configured in such a way as to contain "attractor paths" toward which the subjects' hand paths evolved in the absence of haptic guidance.

**Figure 4:**
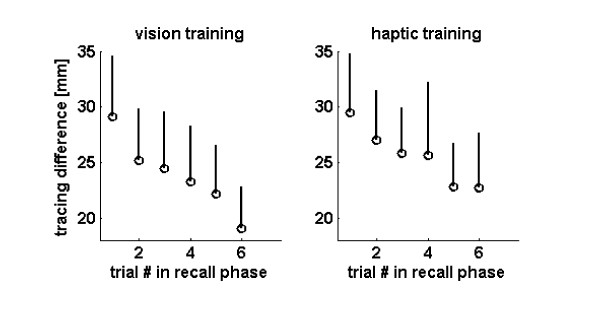
Tracing difference relative to the last path in each recall phase (i.e. recall trial 7) after visual (left) or haptic (right) training. The error bars show one standard deviation across the 20 subjects.

To test this hypothesis, we first compared the tracing error when the last movement (movement 7) of the recall phase was used as the reference. If the hand path evolved systematically toward an attractor path during "forgetting" then this measure should have decreased systematically (as the hand path was drawn toward the attractor path). Figure 4 shows that this was indeed the case, for both visual and haptic training. The tracing error relative to the last reach decreased systematically and significantly during the recall phase (ANOVA, linear contrast, p < 0.001).

**Figure 5:**
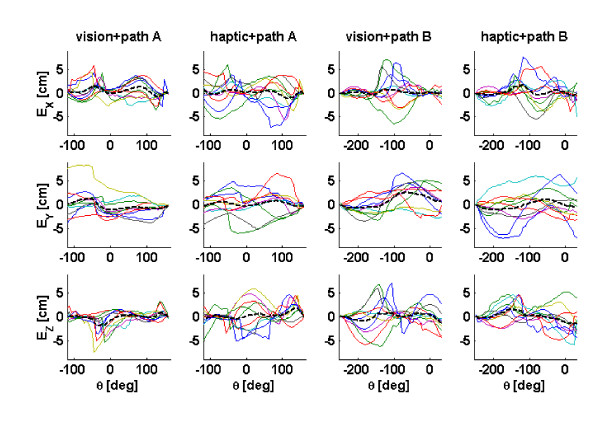
The tracing error in the x, y, and z directions (as defined in Figure 1A) shown as a function of θ, the yaw angle of the path. The last trial (trial 7) in the recall phase is shown for each training condition and each path (vision or haptic training, path A or path B). Each line represents data from one subject, and the thick dashed line is the average of the 10 subjects. Note that groups of subjects exhibited similar spatial patterns in their tracing error, but that not all subjects followed these patterns.

We plotted the differential tracing error on the last recall trial, for the x, y, and z directions to examine if all of the subjects tended toward making errors in the same direction during recall (Figure 5). We found that groups of subject tended to generate errors in the same directions at the same locations along path, but not all subjects followed these group patterns. The average tracing error in each direction was approximately zero, indicating that the subjects did not simply lower their arms or shift their arms left or right.

## Discussion

The main results of this study are, first, both visual demonstration by a robot and haptic guidance with vision allowed healthy subjects to improve their ability to reproduce a novel, desired path that required multi-joint coordination of the arm. The addition of the haptic input to the visual input during the haptic guidance protocol did not significantly improve learning compared to the visual input alone; in fact, visual training was marginally better. The subject's performance significantly decayed over the course of a few movements without guidance. This forgetting process was consistent with the subjects' hand path evolving away from the desired path and toward an attractor path.

### Role of haptic and visual training in trajectory learning

Both repeated haptic guidance and visual demonstration gradually improved the subjects' ability to trace the desired path, with performance improving in a linear-like fashion over the course of 126 movements, or about 20 minutes of practice. These results support the use of haptic guidance or visual demonstration by robotic devices for teaching desired movements. The form of haptic guidance used here was to propel the subject's hand along the desired path. A pilot study for the present study showed that haptic training using a virtual channel that constrained the hand movement but did not propel the hand could also improve tracing performance [28].

This result is consistent with the study of Feygin et al. 2002 [7], which found improvements with haptic guidance or visual demonstration, with a protocol with some differences from the present study. Feygin et al. 2002 evaluated haptic guidance that propelled hand movement using three training techniques including vision only, haptics only, and vision plus haptics, and two recall techniques: attempting to reproduce the movement with vision and without vision. The present experiment consisted of a subset of two of these training techniques – vision only and vision plus haptics, and only one recall technique: attempting to reproduce the movement with vision. These training and recall techniques were selected for the present study because many aspects match a typical rehabilitation situation. Another difference was that the desired curve in the present study was much simpler, and we introduced multiple consecutive recall movements, instead of a single recall movement as in Feygin's experiments, to study retention when guidance was withheld. Despite these differences, the present results are consistent with Feygin's study in that they demonstrate that repeated haptic or visual guidance can improve performance by reducing tracing error. In the present study, visual training without haptic input showed a trend towards greater improvement, while no such trend emerged in Feygin's study.

A possible reason that visual training was marginally better than haptic training is that visual sensation is more accurate than haptic sensation, and thus haptic sensation doesn't improve performance when both types of feedback are available at the same time. In the Feygin 2002 experiment [7], the performance metric for shape learning with haptic information alone was significantly worse than learning with visual information alone, when visual information was available during recall. This suggests an advantage to visual information alone, with the use of these two sources of information not equal. Further, information derived from visual and haptic sensory channels may conflict with each other. Recall with vision was worse than recall without vision following haptic training. Thus, the addition of vision to the recall task in some way degraded performance of the task following haptic training.

Other studies have found that haptic shape information is distorted. For example, Fasse et al. 2000 [29] found that subject's haptic perception of corners was distorted. Henrique and Soechting (2003) [30] showed subjects' perception of a polygon's shape, learned with haptic guidance alone, was significantly distorted from the actual shape. With their eyes closed, subjects had systematic error after they moved the robot along a curved or tilted virtual wall and judged its direction, curvature, relative curvature, rate-of-change-of-curvature, and circularity in different workspaces. In the present study, it may be that the addition of haptic information did not reinforce the internal representation of the desired path because the haptic representation of the path was distorted compared to the subject's visual representation of the path. Furthermore, when visual information is available, even if it is inconsistent with haptic information, several studies have found that it still drives motor adaptation during arm movements [31,32]. In addition, visual presentation of a desired tapping sequence [33], drawing direction for a shape [34], or even visual presentation of the process of learning to adapt to a force field [35] can aid subjects in learning these tasks, again indicating the sufficiency of visual information to drive motor learning.

For some movement tasks, active movement by the subject during training produces more brain activation and better motor learning than movement that is passively imposed on the subject. 2005). For example, Lotze et al. [36] trained subjects to make wrist flexion and extension movements at a desired velocity, while Kaelin-Lang et al. [37] trained subjects to make fast thumb movements in a desired direction. Both studies found that subjects learned the task better when they made the practice movements themselves, as compared to receiving an imposed demonstration of the movement while they remained passive. In contrast, in the present study, active movement by the subject during the haptic training did not substantially improve learning of the trajectory, compared to simply watching the trajectory. The difference of this finding in comparison to these previous findings may be due to the nature of the task studied, due to the decreased errors allowed by haptic guidance, or an indication that visual demonstration of a desired movement is a powerful drive for learning, even if the subject does not move actively during that demonstration. Mirror neurons that discharge similarly during either the execution or observation of hand movement are a possible substrate for this demonstration drive [38]

### Systematic error, forgetting, and attractor paths during robot-assisted trajectory learning

Another interesting finding was that the tracing error increased over the course of several trials when robotic guidance was withheld. The phase of training did not reduce the amount of forgetting: forgetting occurred both early and late in training, although the starting error from which forgetting commenced was smaller later in training (Fig 3).

The changes in the recalled path were not random, but instead were consistent with a systematic evolution toward another path. As mentioned above, systematic distortions in the haptic perception of geometry have been observed previously, with subjects "regularizing" shapes to make them more symmetrical [39,40]. We speculate that the motor system is configured in such a way to contain "attractor paths". These paths may arise because they correspond to commonly perceived shapes. Alternately, they may minimize effort or smoothness, or perhaps they are a basis set for constructing arbitrary paths. The results of this study suggest that attractor paths can be altered with training, as the hand path on the last reach in each recall cycle got systematically closer to the desired path with training (Fig 3). Thus, one benefit of robot-guided path training may be to produce a slow, persistent alteration in the attractor path.

One practical implication of the finding of rapid forgetting is that much of the immediate effect of manual guidance may be lost with further, unguided practice, due to an evolution toward "default modes of moving" (i.e. attractor paths). Devising strategies to reduce forgetting, and thus maximize retention, and to shape attractor paths, are important goals for future research.

### Applicability to rehabilitation therapy

Although the present study focused on healthy subjects, it is relevant to movement training following neurologic injury such as stroke. The finding that the motor system is normally capable of interpreting either visual demonstration or haptic guidance with vision in order to improve motor performance suggests that there will likely be at least some residual ability to learn from both techniques following incomplete neurologic injury. In other words, if some normal motor learning processes are intact, which has been demonstrated following stroke, for example [41,42], and efferent pathways are sufficiently preserved to allow arm movements, then the present study suggests that robot-assisted haptic guidance or visual demonstration can be used to learn new trajectories, or to improve pathological ones, with comparable effectiveness. Specific neurologic impairments might alter this conclusion. For example, damage to visuo-perceptual brain areas may make visual demonstration less effective; in this case, haptic guidance may be particularly useful for training movements. On the other hand, we hypothesize that proprioceptive deficits will not hinder learning from either robot-assisted visual demonstration or haptic guidance, as long as the patient has vision of the arm, as it seems that visual information plays a major role in driving trajectory learning.

## Conclusion

In conclusion, the present experiment indicates that visual demonstration was similar, and perhaps marginally better than haptic guidance with vision, in promoting trajectory learning. There might be circumstances where haptic training is nevertheless preferred, for example, for training movements in which vision of the arm is not possible, such as movements behind the body or head. Therapists typically completely constrain the arm configuration during manual guidance, whereas the current study guided only the hand leaving the subject to resolve the joint redundancy, a difference that might be significant. The device that we used for this study was not capable of constraining the arm posture; however, exoskeletal robots suitable for rehabilitation are becoming available that could be used to study this question [6,32,43]. The current study evaluated motor learning and forgetting over a single session. However, rehabilitation therapy is often administered over many weeks. The extent to which current results generalize over this broader temporal window requires further study. Finally, it may be that in some cases the somatosensory stimulation that arises during haptic guidance reinforces cortical plasticity following neurologic injury, promoting functional recovery; however, this intriguing possibility stills remains to be demonstrated.

## Competing interests

The author(s) declare that they have no competing interests.

## Authors' contributions

JL helped to design the experimental protocol, carried out the experimental protocol, performed the data analysis, and drafted the manuscript. SC helped to design the experimental protocol and revised the manuscript. DR helped to design the experimental protocol and revised the manuscript. All authors read and approved the final manuscript.
